# Peritoneal Mesothelioma Diagnosed on Frozen Section During Diagnostic Laparoscopy: A Case Report

**DOI:** 10.7759/cureus.94474

**Published:** 2025-10-13

**Authors:** Mae Visconti, Jenna M Watts, Wade Weston, Jamie F Cimino, Gerald Englund, Mark Jarosz

**Affiliations:** 1 Surgery, Kansas City University of Medicine and Biosciences, Joplin, USA; 2 Obstetrics and Gynecology, Kansas City University of Medicine and Biosciences, Joplin, USA; 3 Obstetrics and Gynecology, Freeman Health System, Joplin, USA

**Keywords:** asbestos exposure, atypical mesothelial cells, d2-40, diagnostic laparoscopy, gynec oncology, hyperthermic intraperitoneal chemotherapy, malignant peritoneal mesothelioma, peritoneal malignancy

## Abstract

Malignant mesothelioma is a rare neoplasm arising from the proliferation of mesothelial cells. When this proliferation involves the lining of the abdomen, the peritoneum, it is referred to as malignant peritoneal mesothelioma (MPM). We present a case of MPM identified during diagnostic laparoscopy, detailing the patient’s symptoms and prior workup that prompted the procedure. This report describes a rare, rapidly progressive case in an elderly female patient, diagnosed via frozen section and immunohistochemistry, who experienced clinical deterioration over 24 days from initial presentation, highlighting both the diagnostic challenges and the aggressive nature of this disease.

## Introduction

Malignant mesothelioma is a rare neoplasm arising from the proliferation of mesothelial cells [1]. Although strongly associated with asbestos exposure, mesothelioma can develop in the absence of known exposure and may involve any mesothelial lining, including the pleura, pericardium, or peritoneum [1]. Malignant peritoneal mesothelioma (MPM) specifically affects the peritoneal lining of the abdomen and accounts for approximately 7-30% of all mesotheliomas, with an incidence in the United States of 1.94 per 100,000 men and 0.41 per 100,000 women [2]. MPM often presents with nonspecific abdominal symptoms, such as distention or pain, which frequently lead to delays in diagnosis [2]. In a study reporting 2,500 mesothelioma cases in males, 7% involved the peritoneum, whereas among 700 cases in females, 18% were peritoneal [3].

Here, we describe a rare, rapidly progressive case of peritoneal mesothelioma in an elderly female patient who deteriorated over 24 days from initial presentation. She presented with nonspecific symptoms, complicating the diagnostic process, and an initial workup was pursued due to suspicion of ovarian carcinoma. The final diagnosis was established through frozen section and immunohistochemistry. This case highlights both the diagnostic challenges and aggressive clinical course characteristic of MPM.

## Case presentation

An 85-year-old female with a past medical history of hypertension was admitted following multiple visits to the ED for generalized weakness. On initial presentation, she was diagnosed with a urinary tract infection and discharged on oral cephalexin (Keflex). She discontinued the antibiotics on day three and re-presented to the ED with nausea, vomiting, and abdominal pain. The patient also reported ongoing suprapubic tenderness, for which she had been taking Tylenol nightly.

Laboratory studies revealed hypercalcemia of unknown origin, accompanied by decreased parathyroid hormone (PTH) and elevated PTH-related peptide (PTHrP), raising suspicion for hypercalcemia of malignancy (Table 1). Physical examination demonstrated bilateral upper and lower extremity edema with significant bilateral toe discoloration, more pronounced on the left, raising concern for pelvic venous obstruction. Abdominal CT revealed diffuse anasarca with ascites concerning for peritoneal carcinomatosis.

**Table 1:** Laboratory results CA, cancer antigen; CEA, carcinoembryonic antigen; PTH, parathyroid hormone; PTHrP, parathyroid hormone-related peptide

Lab	Value	Reference range
Plasma calcium	12.2 mg/dL	8.4-10.2 mg/dL
Ionized calcium	1.82 mmol/L	1.12-1.32 mmol/L
PTH	6.7 pg/mL	7.9-9.0 pg/mL
PTHrP	129.0 pg/mL	11-20 pg/mL
Vitamin D	20.20 ng/mL	30-100 ng/mL
CA-125	142 U/mL	<35 U/mL
CA 19-9	20 U/mL	<34 U/mL
CEA	0.34 ng/mL	0-3.0 ng/mL

**Figure 1:**
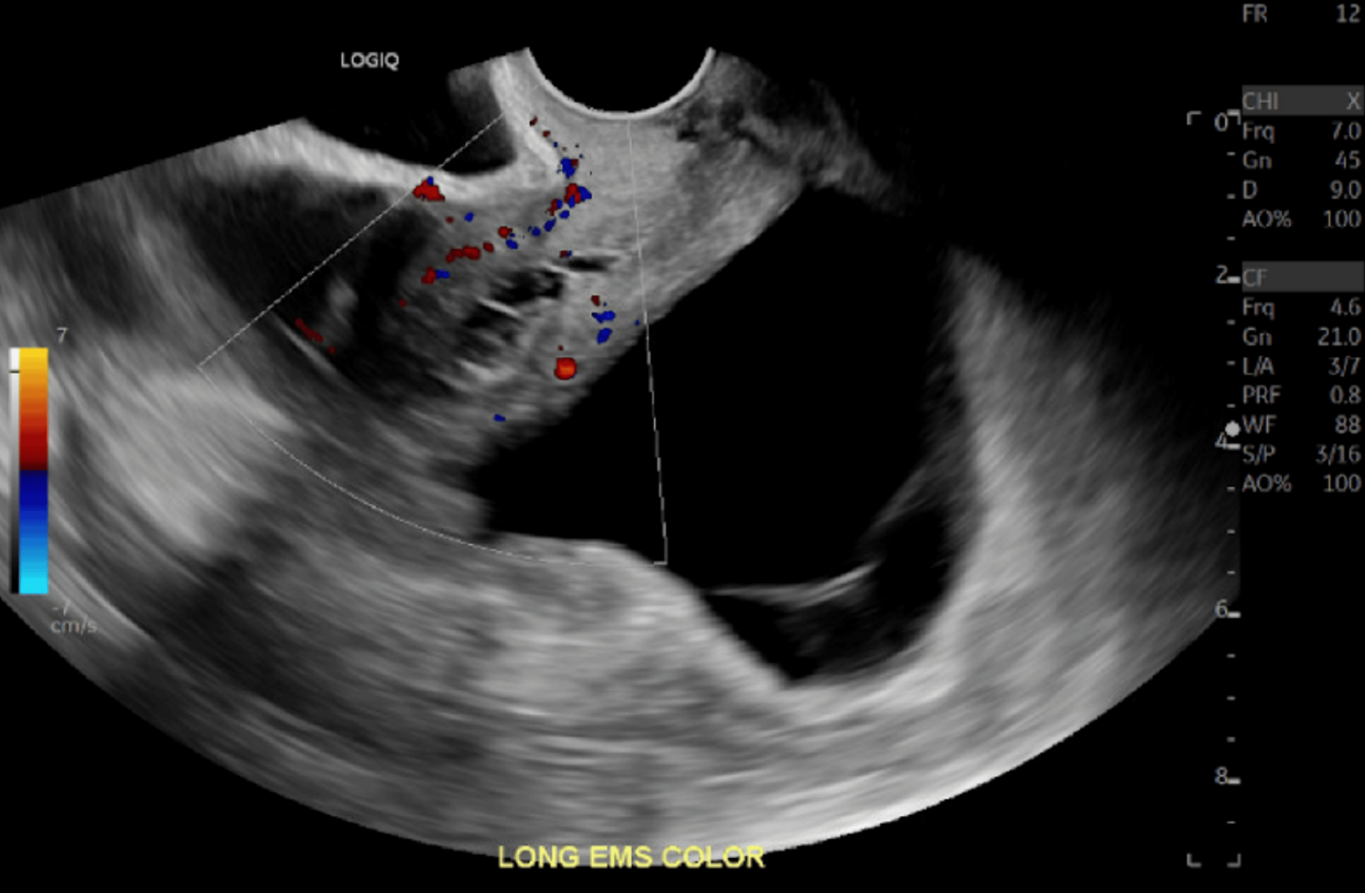
Pelvic ultrasound of the uterus with color Doppler showing a thickened endometrial stripe

**Figure 2:**
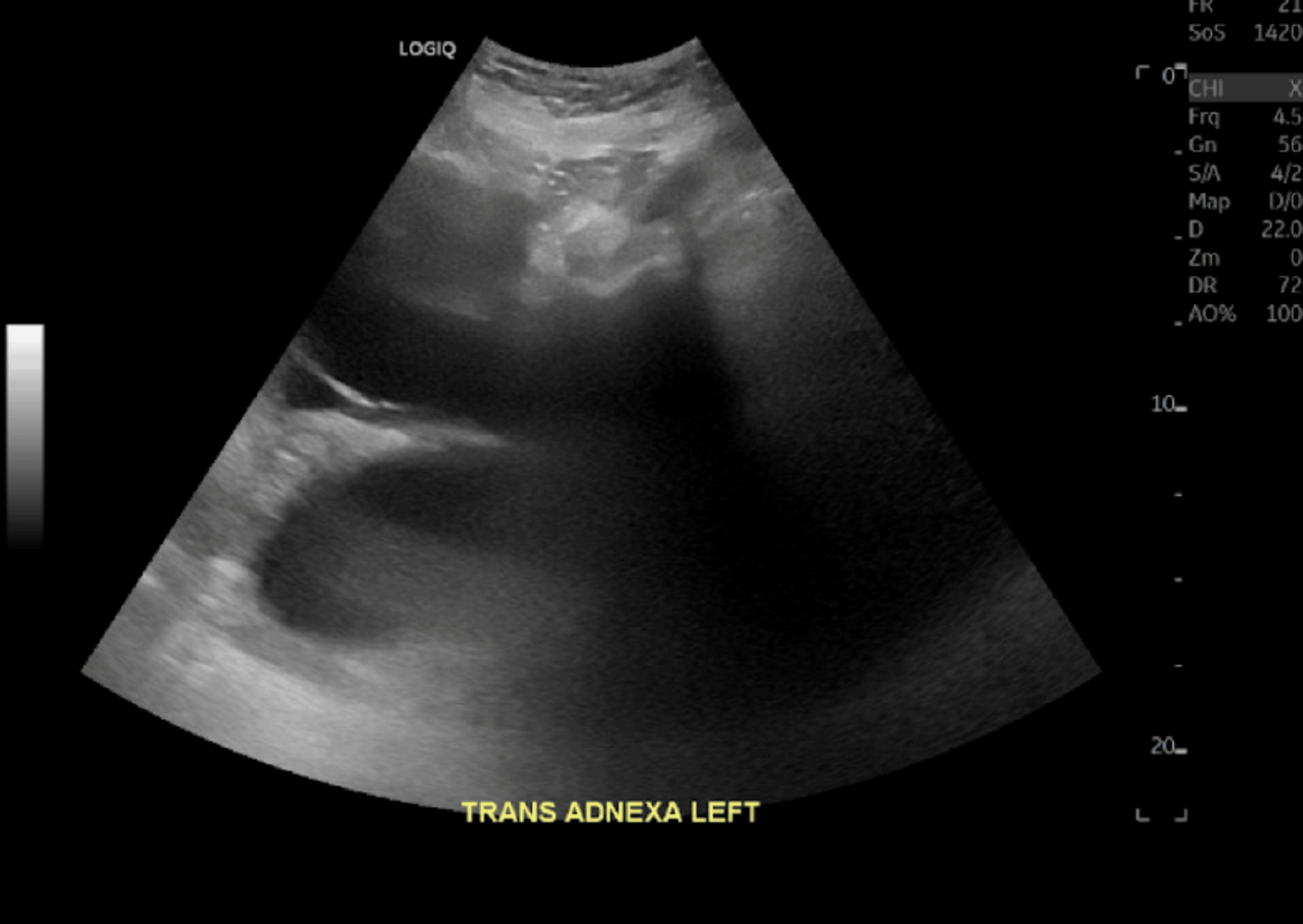
Pelvic ultrasound of the transverse left ovary/adnexa

**Figure 3:**
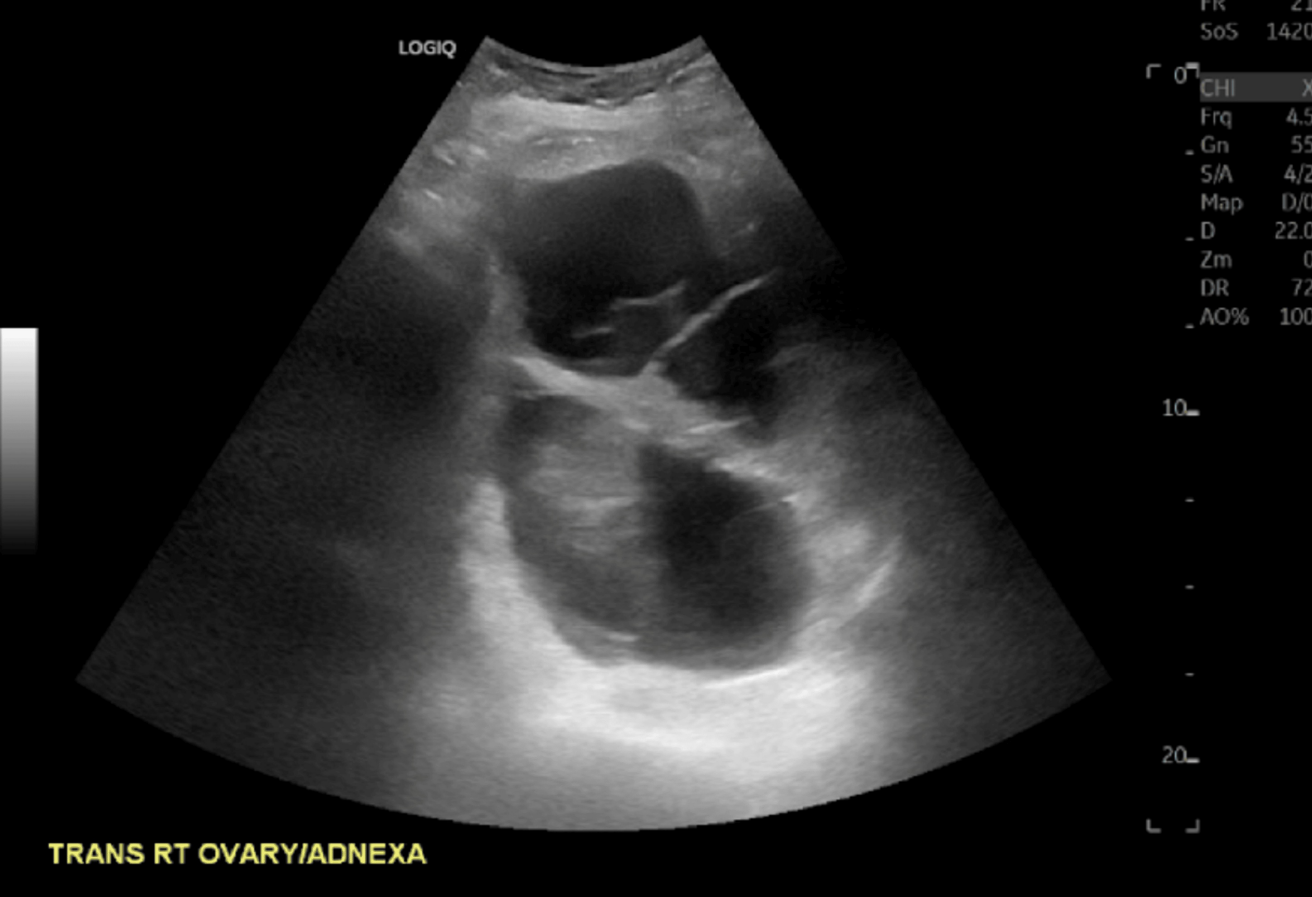
Pelvic ultrasound showing a large multicystic right ovary

Given the physical exam findings, the presence of ascites, and an elevated cancer antigen (CA)-125 of 142 U/mL, the gynecology team suspected ovarian carcinoma and pursued further imaging and a diagnostic laparoscopic examination (Table 1). Ultrasounds demonstrated a normal-sized uterus with a thickened endometrial complex, a poorly visualized left ovary, and a large, multicystic right ovary (Figure 1, Figure 2, Figure 3).

**Figure 4:**
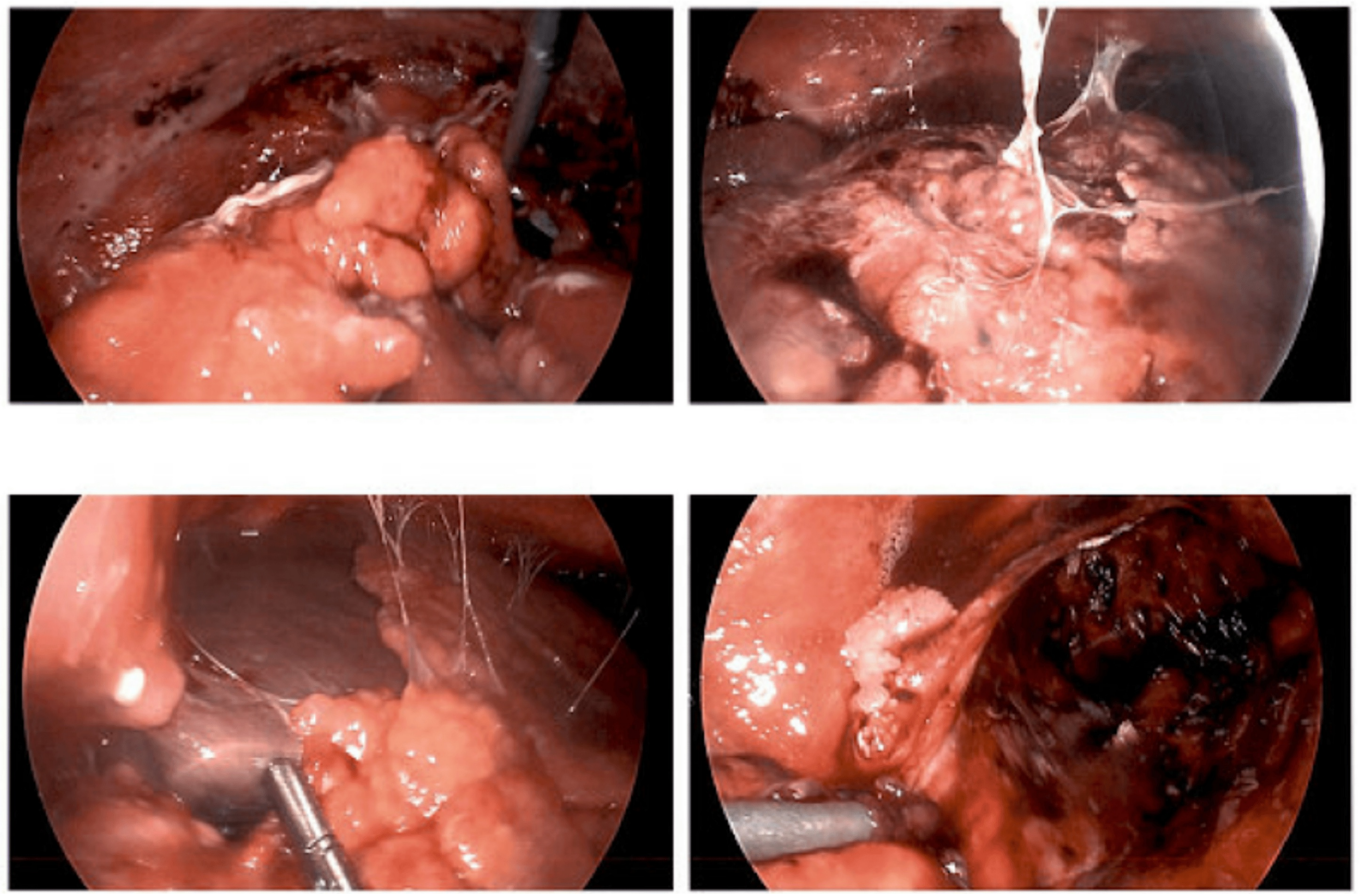
Intraoperative laparoscopic image demonstrating ascites with omental caking

**Figure 5:**
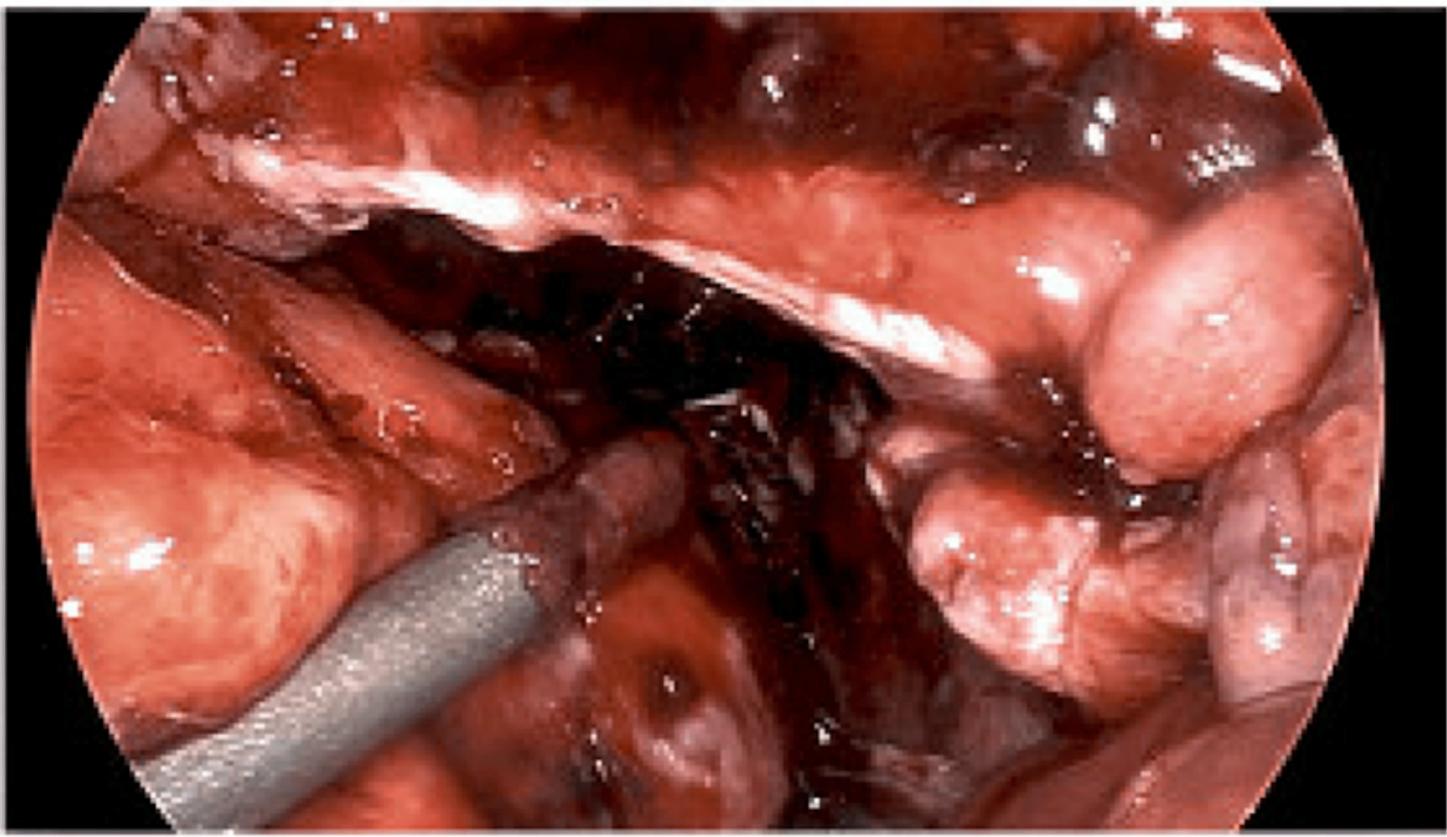
Intraoperative laparoscopic image obtained during diagnostic laparoscopy

Diagnostic laparoscopy revealed omental caking, red-green ascites, and diffuse serosal adhesions (Figure 4, Figure 5). Biopsies of the omentum, uterine serosa, and cul-de-sac were obtained. Due to the abnormal gross findings and concern for tumor seeding, frozen section analysis was performed intraoperatively, revealing atypical mesothelial cells suspicious for peritoneal mesothelioma. 

Further evaluation of the specimens at Mayo Clinic, including immunohistochemistry, showed positivity for cytokeratin AE1/AE3 and D2-40, loss of nuclear MTAP staining, and negative staining for GATA-3 and WT-1. Additional stains, including calretinin and CK5/6, were also negative. These findings confirmed a diagnosis of biphasic MPM (epithelioid: 80% and sarcomatoid: 20%).

Unfortunately, given the patient’s advanced age and rapid clinical deterioration, she was transitioned to in-hospital comfort care and passed away within four weeks of her initial presentation.

## Discussion

MPM is a rare malignancy, accounting for only a minority of mesothelioma cases. Mesotheliomas are strongly associated with exposure to industrial pollutants, most commonly asbestos, though other risk factors such as radiation and certain mineral exposures may also contribute [2]. MPM often presents with nonspecific symptoms, including abdominal pain, distention, weight loss, and fatigue, frequently resulting in delayed diagnosis [2]. Typically multifocal, tumors initially form nodules before spreading locally [4]. By the time of diagnosis, many patients already exhibit diffuse peritoneal involvement, which is associated with poor prognosis. Median survival for MPM is approximately 24 months, with worse outcomes observed in sarcomatoid and biphasic subtypes [2,5].

Histologically, mesotheliomas are classified into three subtypes: epithelioid, sarcomatoid, and biphasic. Epithelioid is the most common and generally correlates with better outcomes, whereas sarcomatoid tumors are more aggressive with poorer prognoses [4]. Biphasic tumors, as in this case, contain both epithelioid and sarcomatoid components and often behave aggressively due to their highly transformative potential in response to injury [4].

Diagnosis of MPM is typically suggested by CT imaging showing a heterogeneous soft tissue mass with irregular margins [2]. Definitive diagnosis requires a core needle biopsy or biopsy obtained via diagnostic laparoscopy [2]. Common findings include ascites, omental caking, and nodular peritoneal masses, all of which were present in our patient [2]. CA-125 may be elevated in MPM but is nonspecific and has limited diagnostic utility [2]. Immunohistochemical markers aid in diagnosis. D2-40 (podoplanin) is commonly positive in malignant mesothelioma [6]. Loss of MTAP expression is observed, particularly in sarcomatoid subtypes [7]. Broad-spectrum cytokeratin (AE1/AE3) has high sensitivity for mesothelial tumors, especially sarcomatoid types [8]. WT1 is usually positive in mesotheliomas, though rare negative cases, such as ours, have been reported [9]. Calretinin and cytokeratin 5/6 are additional commonly used markers, though they were negative in this patient.

First-line treatment for MPM is cytoreductive surgery combined with hyperthermic intraperitoneal chemotherapy (HIPEC), although not all patients are candidates [2]. Eligibility for HIPEC typically includes age under 70 years; given her advanced age, our patient was not a candidate [10]. Hypercalcemia was noted and ultimately attributed to malignancy, the most common cause of hypercalcemia in hospitalized patients [11]. Hypercalcemia of malignancy can result from ectopic production of PTHrP, which was elevated in our patient, or from extensive tumor burden [12].

This case underscores the importance of including MPM in the differential diagnosis for patients presenting with unexplained ascites and abdominal complaints. It highlights the challenge of diagnosing the disease in a frail, elderly patient, where therapeutic options are inherently limited. Although the patient’s initial presentation to the ED involved nonspecific symptoms, she reported ongoing suprapubic tenderness managed with daily Tylenol. A thorough reproductive history and earlier workup might have led to an earlier diagnosis. While earlier recognition may not have changed the outcome, it emphasizes the need to tailor diagnostic intensity to patient frailty and goals of care. Clinically, this case illustrates the value of prioritizing laparoscopy over serial imaging in patients with progressive symptoms and non-diagnostic radiologic findings; early minimally invasive evaluation can provide diagnostic clarity and expedite care decisions [13].

## Conclusions

MPM, though rare, should remain in the differential diagnosis for elderly patients presenting with ascites and abdominal complaints. Early diagnosis is critical but challenging due to the nonspecific nature of presenting symptoms. Maintaining a high index of suspicion and conducting a thorough workup are essential. Prompt gynecologic and surgical evaluation, coupled with timely biopsy and immunohistochemical analysis, is crucial for accurate diagnosis. This case highlights the rapid progression and poor prognosis of MPM, emphasizing the importance of vigilance, comprehensive evaluation, and early diagnostic intervention.
